# Basic suture technique: Instructional videos explaining suturing for medical students in a qualitative study

**DOI:** 10.1016/j.amsu.2019.10.027

**Published:** 2019-11-05

**Authors:** Swapnil D. Kachare, Sara R. Abell, Milind D. Kachare, Joyce Jhang, Bradon J. Wilhelmi, Morton L. Kasdan

**Affiliations:** a Division of Plastic and Reconstructive Surgery, Department of Surgery, University of Louisville, Louisville, KY, United States; b School of Medicine, University of Louisville, United States; c Department of Surgery, Robert Wood Johnson Medical School, New Brunswick, NJ, United States; d Department of Surgery, East Carolina University, Greenville, NC, United States

**Keywords:** Suture, Technique, Instruction, Eversion, Inversion, Skin

## Abstract

**Background:**

Understanding basic surgical skills is important for medical students prior to entering residency regardless of future specialty. In these videos we provide instruction for suturing as it relates to skin closure.

**Material and methods:**

Instructional videos were created by the senior faculty (R.A. and M.K.) to teach medical students at the University of Louisville suturing techniques.

**Results:**

Entering and exiting the needle at an angle of 90° or greater allows for tissue eversion. Inadequate eversion of tissue or inadequate angling of the needle will lead to tissue inversion. When suturing uneven edges, a deep bite on the low side and a shallow bite on the high side will allow for appropriate tissue leveling. For buried sutures, skin eversion with substantial dermal bites and proper knot location is essential.

**Conclusion:**

Understanding the basics of skin apposition will provide students with knowledge about primary wound healing and prepare them for residency.

## Highlights

• Learning suture skills early in medical school leads to future operative confidence.
• The angle of the needle entering the skin determines the level of skin apposition.
• High and low edges of a wound can be leveled with proper suture technique.
• Proper technique can achieve a buried knot in the deep layer of the dermis.

## 1 Introduction

There are many determinants of success in surgery that lead to operative confidence, one of which is the level of technical skill [1]. A challenge for both medical students and residents is acquiring proficiency in suturing. Learning the fundamentals of surgical technique early leads to success on clinical rotations during the third and fourth years of medical school [2]. The use of instructional videos has been shown to aid in teaching students proper suturing techniques, but currently there is no consensus on a standardized video to use [3]. The goal of these videos is to instruct students on a clear and easily replicable method of suturing.

## 2 Methods

For more than 20 years the senior author, a Plastic Surgeon, has conducted weekly Sunday morning classes for students at the University of Louisville School of Medicine. During these classes, students learn how to tie knots and suture prior to beginning their third-year clinical rotations. Enrollment in these sessions were also open to residents, nurse practitioners, and physician assistants. Participation was voluntary and the classes were completely filled every year. Over 900 students took part in the course during the 20 year period, with a total of 162 students between April 2016 to April 2018, who stated a subjective improvement in their suturing skills.

There were four 2 hour sessions, and each class started with a short instructional video prior to hands-on practice using a suture practice board [4]. Each class had a different objective and focused on different aspects of knot-tying and suturing. These instructional videos were created by the two senior faculty (M.K. and R.A). In the video, “Basic Suturing,” the concepts of eversion, inversion, and careful approximation of high and low edges are presented. In the video, “Passing Buried Suture,” both animated and cadaveric demonstrations of buried knots are shown. The cadaveric demonstration portion was filmed in the Fresh Tissue Lab at the University of Louisville School of Medicine, Louisville, KY. The interpretation and description of the data was performed using guidelines provided within the consolidated criteria for reporting qualitative research (COREQ) checklist as described by Tong and colleagues [5].

## 3 Results/findings

### 3.1 Even apposition (Video 1)

There are two key factors when the desired result of tissue closure is even apposition. The first factor is the amount of tissue eversion, and the second is the angle at which the needle is passed relative to the plane of the tissue. For even apposition and eversion, the needle should be passed at a 90° angle to the skin.

Supplementary video related to this article can be found at https://doi.org/10.1016/j.amsu.2019.10.027.

The following is/are the supplementary data related to this article:

Passing buried suture - Video 2.mp4

### 3.2 Eversion (Video 1)

If the desired result of tissue closure is eversion of the skin, the tissues must achieve greater eversion when compared to creating even apposition of tissues. In order to accomplish this increased degree of tissue eversion, the first needle pass should be taken at an angle greater than 90° relative to the tissue plane before repeating the same on the opposing side. A greater angle of needle entrance corresponds to a greater amount of tissue eversion. Therefore, the angle of the needle and degree of tissue eversion are directly correlated, and the wound edges will evert once the knot is tightened.

### 3.3 Inversion (Video 1)

Inversion of the skin should be avoided. Mistakes arise when the tissue is not everted enough or the needle is not rotated back far enough, resulting in a shallow bite and tissue inversion.

### 3.4 High and low edges (Video 1)

High and low edges occur when unequal bites are taken from opposing sides of the tissue. If a shallow bite is initially taken, followed by a deeper bite on the opposing tissues, a high and low edge will be created. This unequal tissue incorporation will pull the deep side up while pulling the shallow side down when the knot is tightened. However, this method is desired when a wound has a pre-existing high and low side prior to suturing. In these cases, taking a deep bite of the low side and a shallow bite of the high side will result in a wound with an even apposition.

### 3.5 Buried knots (Video 2)

In order to create an intradermal buried-knot, several important steps need to be followed. First, the nearest skin edge is everted in order to expose the dermis without directly grasping the skin edge. Next, the needle should be inserted well into the deepest dermal layer in order to take a substantial bite. Further evert the skin and bring the needle out just below the surface of the skin, traveling deep to superficial. On the opposing side, the dermis should be grasped and everted, with the needle inserting just beneath the surface of the skin and exiting at the deepest dermal layer resulting in a superficial to deep trajectory. This method results in a buried knot at the deepest layer of the dermis. For this method to be successful, the needle and the thread must be on the same side of the loop of the inverted “U” stitch. Otherwise, the knot will rest on the loop just beneath the surface of the skin, rather than in a buried position. Finally, as the knot is being tied, it should be tightened along the long axis of the wound, rather than perpendicular in order to ensure the knot remains in the deeper space.

In the cadaveric demonstration, the surgeon showcases smooth, efficient motion, with no wasted movements. An important observation is that the forceps never grasp the surface of the skin, minimizing tissue trauma.

Supplementary video related to this article can be found at https://doi.org/10.1016/j.amsu.2019.10.027.

The following is/are the supplementary data related to this article:

Basic suturing - Video 1.mp4

## 4 Discussion

A proper understanding on the basics of suturing and tissue handing is necessary to skillfully approximate skin [1]. Showing these instructional videos prior to learning in a hands-on session provides students with a visualization of how to evenly appose or evert tissue. There are certain limitations to this study, notably limited student feedback. Further analysis of our data and a student survey study will address this limitation moving forward. However, these videos provide medical students with a starting point to gain the fundamental skills necessary to master the art of basic suturing.

## Sources of funding

Nothing to declare.

## Ethical approval

Nothing to declare.

## Research registration number

Nothing to declare.

## Trial registry number

Nothing to declare.

## Author contribution

Bradon J. Wilhelmi MD: writing.

Milind Kachare: writing.

Sara Abell: writing.

Joyce Jhang: writing.

Morton Kasdan, MD: study design & writing.

## Guarantor

Swapnil Kachare.

## Provenance and peer review

Not commissioned, reviewed by editor(s).

## Declaration of competing interest

Nothing to declare.
